# Smokeless Tobacco and Its Adverse Effects on Hematological Parameters: A Cross-Sectional Study

**DOI:** 10.1155/2019/3182946

**Published:** 2019-04-01

**Authors:** Anjani Kumar Shukla, Tanya Khaitan, Prashant Gupta, Shantala R. Naik

**Affiliations:** Department of Oral Medicine and Radiology, Dental Institute, Rajendra Institute of Medical Sciences, Ranchi 834009, India

## Abstract

**Background:**

Smokeless tobacco (SLT) as a drug substance has been used throughout the world although it has dangerous effects on human health. Among the 28 known carcinogens in SLT, tobacco-specific nitrosamines are considered to be the most potent. This has challenged the metabolic condition leading to a rise in the inflammatory status, increased apoptosis, and red blood cell (RBC) membrane damage. Therefore, the present study was undertaken to evaluate the adverse effects of SLT on hematological parameters and establish a correlation between them.

**Materials and Methods:**

A total of 100 subjects (50 SLT users and 50 nonusers) were selected for the study. Complete demographic data and history were taken and clinical examination was done to evaluate any oral mucosal changes. Venous blood samples were taken to analyze the hematological parameters.

**Results:**

Significant changes were observed in the complete blood profile in SLT users when compared to nonusers. All the hematological parameters had a negative correlation with form of SLT except for total leucocyte count which had a positive correlation.

**Conclusion:**

The current study confers an imperative role into SLT mediated effects on a complete hemogram and might be beneficial in spreading awareness against its usage. It also serves as a forewarning alarm among the population consuming SLT as an alternative to smoking tobacco.

## 1. Introduction

Oral cancer, a modern epidemic among the noncommunicable diseases, is a major problem in the Indian subcontinent where it ranks among the top three types of cancer in the country. 20 per 100,000 individuals are affected by oral cancer accounting for about 30% of all types of cancer in the country. The global burden of cancer continues to increase mostly because of increase in habits of tobacco, particularly smoke and smokeless forms [1].

Smokeless tobacco (SLT) is used in various forms in India such as pan (betel quid) with tobacco, zarda, pan masala, khaini, areca nut and slaked lime preparations, mawa, snuff, mishri, and gudakhu. In addition to the locally prepared products, recently many commercially packed products have been marketed at affordable prices and are easily accessible to everyone, particularly the young and poor [2]. The major tobacco alkaloid nicotine and its principal metabolite cotinine are metabolized to pyridine-N-glucuronides, nicotine-N-Gluc, and cotinine-N Gluc in the liver [3]. Besides the toxic chemicals like polycyclic aromatic hydrocarbons, nitrate, nitrite, nicotine, and acrolein, chemicals such as crotonaldehyde, formaldehyde, and acetaldehyde have also been reported in SLT [4].

According to the World Health Organization, nearly 1/3rd of the global adult populations are tobacco users. Global Adult Tobacco Survey-2 (GATS-2) reports that 28.6% of the population consumes tobacco in any form, 10.7% smoke, and 21.4% use SLT [5]. The prevalence of SLT use is higher among men (27%-37%) compared to women (10%-15%) [6]. As per GATS (2009-10), the prevalence of SLT in India ranges from 5% in Himachal Pradesh and Goa to nearly 50% in Bihar, Jharkhand, and Chhattisgarh [7].

Various pharmacological actions of nicotine and additives and their wide use in many regions and countries may affect the status of hematological parameters and further delineate the effects of tobacco use to systemic health. SLT products act locally on keratinocyte stem cells and are absorbed and act in many other tissues in the body. They produce DNA adducts, principally O-6-methyl-guanine and interfere with the accuracy of DNA replication and mutation, further contributing to the molecular chain of events leading to the malignant transformation of a cell. SLT products modulate the metabolic pattern in a robust way and escalate the risk of systemic inflammation such as RBC morphology modulation, polycythemia vera, and cardiovascular diseases. Indeed chromosomal instability resulting from SLT is most often studied in lymphocytes from peripheral blood [4, 8, 9].

Very few studies on the effect of the consumption of SLT on alteration in the levels of hematological parameters have been reported in the literature but no correlation has been established regarding the same. Considering the above background, the aim and objectives of the present study were to determine the effect of smokeless tobacco on hematological parameters in SLT users and nonusers and evaluate the correlation of smokeless tobacco form with complete blood profile.

## 2. Materials and Methods

A cross-sectional study was conducted at the Department of Oral Medicine and Radiology, Dental Institute, RIMS, Ranchi, on a total of 100 subjects (50 SLT users and 50 nonusers). The participants enrolled in the study belonged to the age group of 20-85 years and were selected through a simple random sampling technique. The refusal rate was found to be 7.4% (8 subjects refused to participate as they did not want to undergo any investigatory procedure) and these subjects were not included in the study. The study was explained to all the subjects and written informed consent was obtained. Demographic data (including occupation and socioeconomic status) was obtained for all individuals.

### 2.1. Inclusion Criteria

Healthy individuals with history of consumption of smokeless tobacco in any form and no history of any systemic illnesses were selected as SLT users (exposed). Age and sex matched healthy individuals with no history of tobacco consumption in any form and no history of any systemic illnesses were selected as nonusers.

### 2.2. Exclusion Criteria

Subjects with any systemic illness or immunocompromised conditions, those consuming alcohol and smoking tobacco in any form, and those not willing to participate were excluded from the study.

The armamentarium consisted of diagnostic instruments, a 5 ml syringe, vials containing ethylenediaminetetraacetic acid (EDTA), a tourniquet, sterile cotton, and surgical gloves. 5 ml of venous blood was collected from all subjects by using a routine venipuncture method and stored in vials containing EDTA. Complete blood count was analyzed using an automated blood cell counter by Horiba XL 80 at the hematology laboratory of the institution.

All the data obtained was noted in a proforma specially designed for the study. Comparison of the various parameters of complete blood profile in SLT users and nonusers was performed using the* t*-test and Spearman's rank correlation coefficient with SPSS (Statistical Package for the Social Sciences) software version 16.01. Significance level was considered at 1% (*p* value <0.01) and 5% (*p* value <0.05).

## 3. Results

A total of 100 subjects, 50 SLT users (48 males and 2 females) and 50 nonusers (46 males and 4 females) with mean ages of 40.2 years and 40.5 years, respectively, were selected for the study.  Table 1 represents the detailed sociodemographic characteristics of SLT users and nonusers including occupation and socioeconomic status.

In terms of exposure, consumption of SLT was described in terms of duration (number of months/years of consumption) and frequency (number of times of consumption per day). Among 50 SLT users (exposed), 29 subjects consumed khaini whereas 21 consumed gutkha. When duration of the habit was being compared, the majority of the subjects (24 SLT users) were reported with >10 years, followed by <5 years (15 SLT users) and 5-10 years (11 SLT users). The mean average of duration of khaini consumption was 16.14 years at a frequency of 11 times/day. The mean average duration of gutkha consumption was 6.24 years at a frequency of 6 times/day.

The various oral mucosal changes observed were white lesions (16 SLT users), proliferative/ulcerative growth (17 SLT users), and mixed red and white lesions (2 SLT users). No mucosal changes were observed in 15 SLT users and all the 50 nonusers.  Table 2 shows the distribution of oral mucosal changes seen in SLT users according to duration of consumption. Burning sensation was noted in 26 SLT users.

### 3.1. Comparison of Hemoglobin (Hb %) in SLT Users (Exposed) and Nonusers

**Figure 1:**
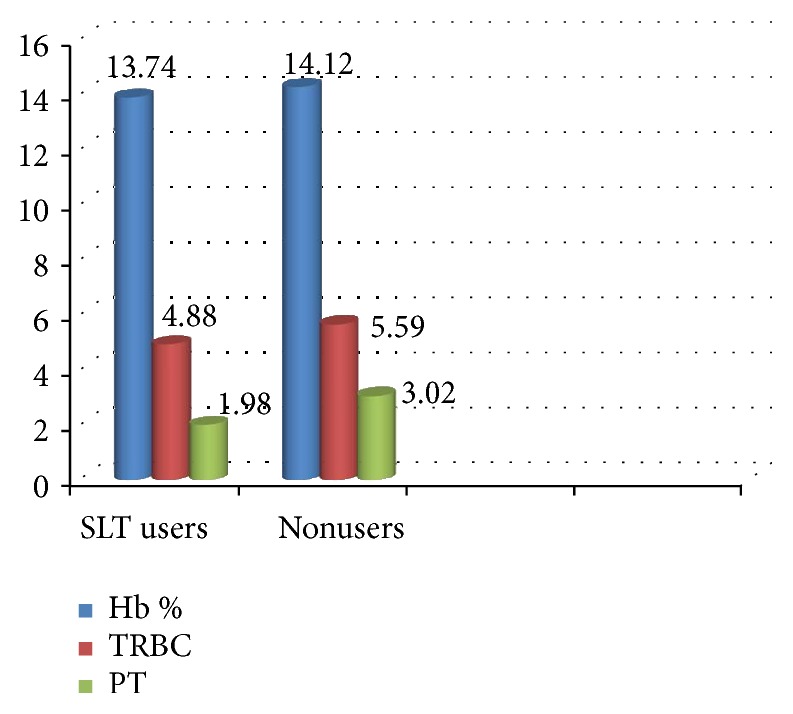
Comparison of Hb %, TRBC, and PT between SLT users (exposed) and nonusers.

Mean serum* Hb*% was lower in SLT users (13.74 g/dl) when compared to nonusers (14.12 g/dl) which was statistically nonsignificant with a* t* value of 1.18 and* p* value of 0.12 (Figure 1).

### 3.2. Comparison of Total Leucocyte Count (TLC) in SLT Users (Exposed) and Nonusers

**Figure 2:**
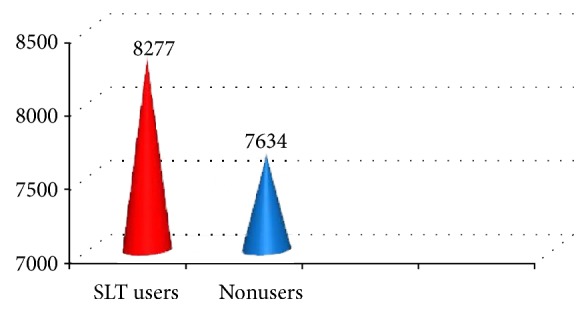
Comparison of TLC between SLT users (exposed) and nonusers.

Mean serum TLC was higher in SLT users (8277 cells/cu.mm) when compared to nonusers (7634 cells/cu.mm) which was statistically significant with a* t* value of -1.66 and* p* value of 0.04 (Figure 2).

### 3.3. Comparison of Total Red Blood Cells (TRBC) in SLT Users (Exposed) and Nonusers

Mean serum TRBC were lower in SLT users (4.88 mill/cu.mm) than nonusers (5.59 mill/cu.mm) which was highly statistically significant with a* t* value of 4.64 and* p* value of 0.00 (Figure 1).

### 3.4. Comparison of Total Platelet (PT) in SLT Users (Exposed) and Nonusers

Mean serum total PT was lower in SLT users (1.98 lakh/cu.mm) when compared to nonusers (3.02 lakh/cu.mm) which was highly statistically significant with a* t* value of 7.16 and* p *value of 0.00 (Figure 1).

### 3.5. Comparison of Mean Corpuscular Hemoglobin Concentration (MCHC) in SLT Users (Exposed) and Nonusers

**Figure 3:**
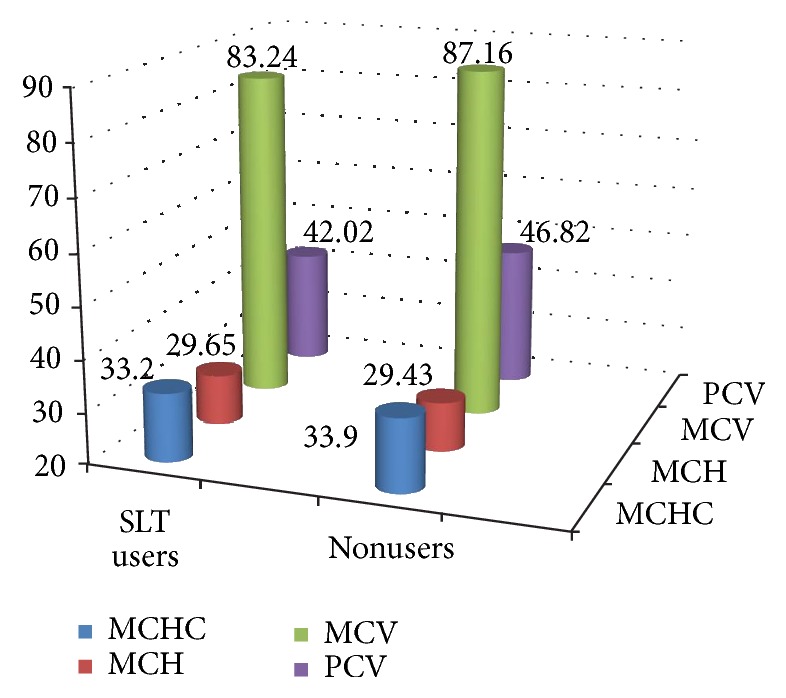
Comparison of MCHC, MCH, MCV, and PCV between SLT users (exposed) and nonusers.

Serum MCHC did not show much difference in SLT users (33.2 g/dl) and nonusers (33.9 g/dl) and was statistically nonsignificant with a* t* value of 2.26 and* p* value of 0.98 (Figure 3).

### 3.6. Comparison of Mean Corpuscular Hemoglobin (MCH) in SLT Users (Exposed) and Nonusers

Serum MCH was higher in SLT users (29.65 pg) when compared to nonusers (29.43 pg) which was statistically nonsignificant with a* t* value of -0.16 and* p* value of 0.43 (Figure 3).

### 3.7. Comparison of Mean Corpuscular Volume (MCV) in SLT Users (Exposed) and Nonusers

Serum MCV was lower in SLT users (83.24 fl) when compared to nonusers (87.16 fl) which was statistically significant with a* t* value of 2.18 and* p* value of < 0.01 (Figure 3).

### 3.8. Comparison of Packed Cell Volume (PCV) in SLT Users (Exposed) and Nonusers

Mean serum PCV was lower in SLT users (42.02%) when compared to nonusers (46.82%) which was statistically significant with a* t* value of 3.35 and* p* value of 0.00 (Figure 3).

### 3.9. Correlation of Form of Smokeless Tobacco with Hematological Parameters

All the hematological parameters had a negative correlation with khaini and gutkha except TLC (positive correlation) and were statistically not significant (Table 3). This was suggestive of the fact that both khaini and gutkha had equally adverse systemic effects on the blood profile.

## 4. Discussion

Tobacco is the dried and processed leaves of the plant* Nicotiana tobacum* that is widely cultivated and commercially grown in many countries of the world [8]. SLT contains three to four times more nicotine than that delivered by a cigarette and it stays for a longer time in the bloodstream. Nicotine is a psychoactive ingredient, metabolically inactivated by CYP2A6 to* cotinine*, metabolized by the liver and detoxified [4].

SLT products commonly observed in our present study were khaini and gutkha. Khaini is prepared from sun-dried tobacco and slaked lime. Gutkha is a ready-to-eat SLT product comprised of areca nut, slaked lime, catechu, tobacco, flavoring agents, and sweeteners. Slaked lime is composed of calcium hydroxide obtained from limestone or sea shells. In addition, it also contains iron, magnesium, and a number of trace elements. The addition of slaked lime and other alkaline agents like magnesium carbonate boosts the pH of a product and results in increased availability of free nicotine, the form that is most easily absorbed. Toxic metals in SLT products include Pb, Cd, As, Cu, Hg, and Se [10].

SLT consumption is more prevalent among lower socioeconomic groups in India that include poor, semiskilled manual workers and unemployed people with meager education. It is believed that the usage of SLT relieves work related stress and has healing properties for curing toothaches, headaches, and stomachaches which forces many adults to accede to its usage. SLT products such as gutkha are considered a form of sweet candy by children and as a mouth freshener by some. Curiosity, peer pressure, and offering by friends and acquaintances contribute to the initiation of its use [11, 12]. Tobacco use is more common among males especially teenagers when compared with females [13]. Male predominance in the age group 30-40 yrs was seen in the present study. The majority of subjects (43 SLT users) were from low socioeconomic background.

The habit of chewing or holding of SLT in the oral cavity also allows absorption of nicotine and other carcinogens through the oral mucosa. This could be the reason behind injury to the oral epithelium caused by tobacco-related toxic products which in turn increases mucosal permeability and penetration of carcinogens. As an early sign of damage to the oral mucosa, chewers of SLT with or without tobacco often develop clinically visible whitish (leukoplakia) or reddish (erythroplakia) lesions and/or stiffening of the oral mucosa and oral submucous fibrosis which later transforms into malignancy. All these oral mucosal changes were observed in the present study [4, 14, 15].

The effects of SLT on physiological systems are also well known. SLT chewers have shown alteration in the morphology of RBCs. Scanning electron microscopy revealed change in RBC membranes with loss of their discoid shape with fine “bubble-like” protrusions. The ingredients of SLT perturb the cellular metabolism of the individuals leading to alteration in shape and size causing enormous consequences in the context of maintaining health [4].

The increase in total RBC count in SLT users seems to reflect that consuming tobacco may also stimulate erythropoiesis. Insufficient pulmonary function in SLT consumers may impart a necessity of stimulating erythropoiesis for fulfilling the oxygen demands of the body. Thus the increase in PCV and Hb levels following such increase in erythrocyte production is quite awaited [8]. Decreases in total RBC, PT, Hb, and PCV levels were seen in SLT users when compared to nonusers and were statistically significant in the present study. Similar decreases in total RBC count, PT, Hb, and PCV levels were seen in the study conducted by Metin et al. (2004) [16]. On the contrary, total RBC, Hb, and PCV levels were increased in the study conducted by Roan Mukherjee et al. (2013) [17].

Nicotine present in tobacco may influence the suprarenal glands to secrete more catecholamine which may affect leukocytosis causing damage to tissue and inflammation [18]. Higher TLC was observed in SLT users when compared to nonusers in the present study and was statistically significant. Similar increase in leukocyte count was also seen in studies conducted by P. Jaganmohan et al. (2011) and Saeed R et al. (2005) [19, 20].

Blood indices such as MCV, MCHC, and MCH relate to individual red blood cells and indicators of anemia. A decreased level of MCV relates to iron deficiency anemia and elevated levels are indicative of anemia of vitamin deficiency [21]. MCHC did not show much difference in SLT users and nonusers and was statistically significant. On the contrary, MCHC count was slightly increased in studies conducted by B. Purushottama Dass et al. (2013) and Biswas et al. (2015) [4, 22]. MCH level was increased in SLT users when compared with nonusers in the present study. Similar increase in MCH level was also present in the studies conducted by B. Purushottama Dass et al. (2013) and Biswas et al. (2015) [4, 22]. MCV level was decreased in the present study. Similar decrease in MCV level was seen in the study conducted by Purushottama Dass et al. (2013) and there was increase in the levels in the study by Biswas et al. (2015) [4, 22]. The altered hematological parameters in SLT users further suggest selective toxicity of SLT and its components.

The noxious components of khaini and gutkha might have an adverse effect on the blood profile. In the present study, all the hematological parameters had a negative correlation with khaini and gutkha and were statistically not significant, indicative of the fact that both khaini and gutkha had equally adverse systemic effects on the blood profile.

## 5. Limitations

Evaluation of all the toxic components present in khaini and gutkha and the differential dosage of these SLT products consumed were beyond the scope of the study. Further studies should be carried out to investigate the prevalence rates of different tobacco products separately along with detailed measures of tobacco consumption, as the economic and health effects of different products may vary considerably.

## 6. Conclusion and Recommendation

SLT use has severe adverse effects on hematological parameters. The present study might serve as an early diagnostic tool in any systemic diseases and be helpful in spreading awareness on the deleterious effect in the populace consuming SLT. Timely intervention among students can prevent the initial experimentations with tobacco from developing into addiction in adulthood. People should be counseled to avoid all habits of tobacco and undergo nicotine replacement therapy along with antioxidants. Knowledge and awareness about systemic and oral ill effects of tobacco should be spread through tobacco control programs in the pursuit for a tobacco-free world.

## Figures and Tables

**Table 1:** Sociodemographic characteristics of SLT users and nonusers.

S. no.	Variable	Frequency	
		SLT users	Nonusers
1	No. of patients	50	50

2	Mean age (years)	40.2	40.5
	Age range (20-85 years)		

3	Gender		
	Male	48	46
	Female	2	4

4	Occupation		
	Employed	13	14
	Self-employed	19	16
	Unemployed	18	20

5	Socioeconomic status*∗∗*		
	Upper	2	1
	Upper middle	8	7
	Lower middle	11	12
	Upper lower	17	16
	Lower	12	14

(*∗∗*: classified according to Kuppuswamy's socioeconomic status scale 2018.)

**Table 2:** Distribution of oral mucosal changes seen in SLT users according to duration of consumption.

Variable	Oral mucosal changes				
	No mucosal changes	White lesions	Mixed red and white lesions	Proliferative/ulcerative growth	Total
Duration					
<5 years	8	4	1	2	15
5-10 years	6	5	0	0	11
>10 years	1	7	1	15	24

**Table 3:** Correlation of form of smokeless tobacco with blood profile.

S. no.	Variable	Spearman correlation (rho)	*p* value
1	TLC	0.01	0.93

2	TRBC	-0.26	0.06

3	PT	-0.09	0.49

4	MCHC	-0.27	0.04

5	MCV	-0.06	0.64

6	PCV	-0.073	0.61

## Data Availability

No data were used to support this study.
